# Abusive use of anabolic androgenic steroids, male sexual dysfunction and infertility: an updated review

**DOI:** 10.3389/ftox.2024.1379272

**Published:** 2024-04-22

**Authors:** Rafael de Almeida Azevedo, Bruno Gualano, Thiago Afonso Teixeira, Bruno C. G. Nascimento, Jorge Hallak

**Affiliations:** ^1^ Applied Physiology and Nutrition Research Group, Center of Lifestyle Medicine, Faculdade de Medicina FMUSP, Universidade de São Paulo, São Paulo, Brazil; ^2^ Division of Urology, Department of Surgery, University Hospital, School of Medicine and Drug Research Laboratory, Federal University of Amapa, Macapá, Brazil; ^3^ Men’s Health Study Group, Institute for Advanced Studies, University of Sao Paulo, São Paulo, Brazil; ^4^ Androscience Institute for Science, Education and Advanced Projects in Male Health, São Paulo, Brazil; ^5^ Division of Urology, Department of Surgery, University of Sao Paulo, São Paulo, Brazil; ^6^ Androscience—Science and Innovation Center and High Complexity Clinical and Research Andrology Laboratory, São Paulo, Brazil

**Keywords:** testosterone, performance enhancement, anabolic androgenic steroids, AAS, andrology, semen, spermatozoa, testis

## Abstract

The evolving prevalence of anabolic androgenic steroids (AAS) abuse among nonathletes is alarming because of the known harm to an individual’s health. Among the adverse effects of AAS abuse, male infertility and sexual dysfunction have been often reported in the literature, but little is known regarding its actual prevalence, possible underpinning mechanisms, and potential treatments either during or post-AAS usage. Thus, the current narrative review summarizes the state-of-art regarding the effects of AAS on male fertility and sexual function. Evidence was gathered from the latest reviews and recent original studies, specifically from prospective cohorts and clinical trials, ultimately resulting in five main topics of discussion. First, AAS usage is briefly characterized by its historical background, main physiological mechanisms, and the most frequently used AAS substances. Second, data on the prevalence of AAS-induced male infertility and sexual dysfunction are described. Third, some new insights on possible underpinning mechanisms of AAS-induced male infertility and sexual dysfunction are thoroughly discussed, with particular attention to histological data derived from animal models and the latest insights from prospective cohorts in humans. Fourth, the potential treatments during and after the AAS usage are presented, highlighting the odds of resolving male infertility and sexual dysfunction. Fifth, future directions on this topic are discussed, focusing on the methodological robustness of scientific studies.

## 1 Introduction

The abusive use of anabolic androgenic steroids (AAS) (defined as a non-therapeutic use, primarily for aesthetic and physical performance purposes) is a silent worldwide problem that negatively affects the health of males and females of various age groups (Pope, Wood, et al., 2014a). The AAS are synthetic and similar to the hormone testosterone, which can be included in the list of performance and image-enhancing drugs (PIEDs) (Pope, Wood, et al., 2014a). The concern regarding abusive use of AAS is justified by the potential severe health harms outcomes, such as the increased risk of sudden death (Montisci et al., 2012). In fact, recent data from a large cohort study in Danish physically active population has shown that all-cause mortality is about two times higher (i.e., hazard ratio of 2.81) in AAS abusers compared to control group (Windfeld-Mathiasen et al., 2024).

Among the adverse effects reported by AAS users, male fertility and sexual function have surfaced as having a steadily progressive high prevalence (de Souza and Hallak, 2011; Rizzuti et al., 2023). Additionally, the fact that relatively young males are the most frequent AAS abusers makes the situation even more alarming, since these individuals are likely to continue the AAS usage throughout their lifespan (Pope, Kanayama, et al., 2014). Nonetheless, alongside the evolving number of AAS users worldwide, the amount of misleading, misguided, and purposeful fake information has also grown regarding the side effects and possible treatments for male infertility and sexual dysfunction induced by AAS abuse. For example, it is not rare to find unsubstantiated use of “nature-based interventions” to treat AAS-induced adverse effects (Santos et al., 2022) and a lack of information from clinicians regarding a practical approach when dealing with AAS users (Eu et al., 2023). Additionally, as the public and academic attention on this topic has sharply grown in the last decade or so, new scientific information is available regarding the prevalence of AAS effects on male fertility and sexual function, possible underlying mechanisms, and treatments.

This narrative review aims to summarize up-to-date evidence about abusive use of AAS on male infertility and sexual dysfunction. The most relevant manuscripts on the topic were searched on MEDLINE and Web of Science. The searching strategy used the terms “anabolic androgenic steroids” combined with “male sexual function,” “male sexual dysfunction” or “male infertility.” Overall, the state-of-art information was organized into five topics. First, AAS usage is briefly characterized in terms of its historical background, main physiological mechanisms, and the most frequent AAS substances currently used. Second, data on the prevalence of AAS-induced male infertility and sexual dysfunction are described. Third, new scientific insights on possible underpinning mechanisms of AAS-induced male infertility and sexual dysfunction are thoroughly discussed, with particular attention to histological data derived from animal models and new findings from prospective cohorts in humans. Fourth, the potential treatments during and after the AAS usage are presented, highlighting the odds of resolving male infertility and sexual dysfunction. Fifth, future directions on this topic are discussed, focusing on methodological robustness of the current findings. We expect this narrative review may help to inform prevention, treatment, and a new research and clinical agenda on male infertility and sexual dysfunction induced by AAS abuse.

## 2 Brief background and current characteristics of AAS use

Historically, AAS abuse has been focused on synthetic testosterone, which was isolated and artificially synthesized in 1935 (Corona et al., 2014), and later utilized by German soldiers during World War II (Pope, Wood, et al., 2014b). Two decades later, in 1956, the first testosterone abuse in sports was reported by Russian weightlifting athletes at Vienna Olympic Games (Pope, Wood, et al., 2014b), but only by 1975 the International Olympic Committee created a list of banned illicit substances, which included a wide range of AAS substances (Anawalt, 2019). Despite growing surveillance from the World Anti-Doping Agency (WADA), the use of AAS is an evolving silent issue in sports, and non-sports environments. For example, in a relatively recent dataset from WADA, it was revealed that up to 70% of professional athletes had declared past use of illicit drugs, including AAS substances, even though only less than 1% was caught (de Hon et al., 2015). It must be highlighted that the majority (∼75%) of AAS users are noncompetitive athletes (Pope, Wood, et al., 2014b; Sagoe et al., 2014; Anawalt, 2019), in which there is a prevalence of AAS use ten times higher in males compared to females (Sagoe et al., 2014). Overall, it has been estimated that the global prevalence of AAS use is 6.4% among physically active males (Sagoe et al., 2014), a prevalence similar to other drugs, such as cannabis and opioids (World Drug Report, 2023). Moreover, the prevalence of AAS users can reach up to 30% depending on the country, region, and sports practice environment (Abrahin et al., 2014). For example, the number of AAS abusers are rare in Eastern Asia but high in Brazil, Commonwealth countries, Europe and North America (Pope, Wood, et al., 2014b). Of relevance, approximately 32% of AAS users can be considered addicted (i.e., continuous or frequent users) (Pope, Kanayama, et al., 2014), as most users are affected by post-use withdrawal syndrome, which is characterized by symptoms of depression, a marked drop in libido and anhedonia (Pope, Wood, et al., 2014b; Anawalt, 2019) ultimately leading to continuous and recurrent use of AAS.

Testosterone and its derivates are mostly used because of their androgenic and anabolic effects in the male body (de Souza and Hallak, 2011; Bond et al., 2022). Specifically, testosterone is a relevant hormone during males’ puberty, as it promotes hair growth, sebaceous gland activity, sperm maturation, and libido (de Souza and Hallak, 2011; Bond et al., 2022). The anabolic effects of testosterone derive from increased protein synthesis and diminished protein degradation (Sjöqvist et al., 2008), while it also increases lipolytic activity (Kadi, 2008), thereby reducing body fat percentage and augmented lean body mass. Briefly, the mechanisms by which testosterone acts in the body depends on its attachment to androgenic receptors (AR) in targeted tissues; also, testosterone can bind to AR receptors when reduced to 5α-dihydrotestosterone (5DHT). Finally, testosterone may be aromatized to oestradiol, regardless if under AAS abuse or in normal physiological concentrations, to exert estrogenic effects, such as water retention, breast tissue growth, and increased fat deposition (de Souza and Hallak, 2011; Bond et al., 2022).

As a neuroendocrine hormone, testosterone is part of the hypothalamic-pituitary-gonadal axis (HPG), which also involves the luteinizing hormone (LH) and follicle-stimulating hormone (FSH) (de Souza and Hallak, 2011; Bond et al., 2022). The hypothalamus secretes gonadotropin-releasing hormone (GnRH) that stimulates the secretion of FSH and LH in the pituitary gland, ultimately leading to gonadal secretion of either testosterone in males or oestradiol and progesterone in females. Notably, the HPG axis is fine-tuned by negative feedback according to the circulating testosterone levels. Thus, the higher the circulating testosterone levels in the body, the lower the activity of the HPG axis, ultimately leading to the cessation of endogenous production of FSH, LH, and testosterone (de Souza and Hallak, 2011; Bond et al., 2022). Daily testosterone synthesis ranges from 2.1 to 11.0 mg in young males, with normal plasmatic levels varying between 300– and 1,000 ng/dL, “with a progressive decline” in its synthesis as a function of aging (de Souza and Hallak, 2011; Bond et al., 2022). However, high AAS doses (e.g., ≥200 mg per week) are associated with decreased endogenous testosterone production and spermatogenesis, as well as gonadal atrophy (Nieschlag and Vorona, 2015). Of note, other substances, such as 25-hydroxy-vitamin D_3_ [25(0H)D_3_], are likely to exert a positive effect on testosterone levels and semen quality (Ciccone et al., 2021). Nonetheless, the abusive and prolonged use of AAS can negatively affect several health parameters, possibly resulting in male infertility and sexual dysfunction (de Souza and Hallak, 2011).

Overall, the usage of testosterone and other AAS substances aims for diminished androgenic effects while enhancing anabolic results. For this reason, there are different AAS compounds and forms of usage to maximize lean body mass and blunt any side effects, such as gynecomastia, acne, and general health problems elicited by high levels of circulating testosterone in the body [i.e., for details, see Bond et al. (2022)]. Briefly, the most common routes of administration are either oral or intramuscular injection, the latter promoting greater bioavailability (Bond et al., 2022). In terms of AAS substances, nowadays, there is a wide range of synthetic derivates of testosterone, which can be classified according to their primary effects, such as 1) testosterone-like effect (e.g., cypionate and undecanoate), 2) DHT-like effect (e.g., stanozolol and oxandrolone), and 3) nandrolone-like effect (e.g., trenbolone and nandrolone decanoate). For details about the routes of administration, chemical structure, and specific effects of each type of AAS, the reader is referred to recent reviews on the topic (de Souza and Hallak, 2011; Bond et al., 2022; Rizzuti et al., 2023).

Most of the evidence about the effects of AAS on urological and andrological parameters comes from controlled environmental settings, such as administering a single AAS compound under a known dosage (Bond et al., 2022). However, the abovementioned situation does not reflect the reality of most AAS abusers, who usually adopt a polypharmacy strategy to maximize physical performance gains while counteracting any harmful and undesired aesthetic side effects (Smit et al., 2020; Bond et al., 2022; Smit et al., 2022). For example, recent findings (Smit et al., 2020) have demonstrated that AAS users may utilize a median of five different AAS substances during the cycle (i.e., period of AAS usage), with a range of one to eleven different substances, totaling a median of 901 mg of testosterone per week (i.e., range 250–3,382 mg) over 13 weeks of AAS-cycle duration (i.e., range 2–52 weeks). Additionally, the most utilized compound, as stated on the label, was testosterone (96% of the users), followed by trenbolone (52%), drostanolone (39%), and boldenone (38%) (Smit et al., 2020). It must be highlighted that the majority of AAS users acquire their substances through the parallel market, which understandably is characterized by no quality-control standards as those available in pharmacies or provided by the pharmaceutical industry. For example, in the abovementioned study, it was found that 47% of the AAS samples analyzed contained undeclared substances, 35% had declared and undeclared substances, and only 13% of the samples matched the label stated by the sealer (Smit et al., 2020). Of note, the remaining 5% of the analyzed samples did not contain AAS substances. Attention should also be taken to dietary supplements (e.g., in the format of powders, pills, gels, bars, shakes, and/or liquids) commercialized through different distribution channels (e.g., supermarkets, gyms, hypermarkets, nutritional and “natural products” stores, drugstores, and/or online). These supplements may contain AAS substances not listed on their label, such as: pro-androgenics, xenoestrogens, estrogens, selective androgen-receptor modulators, nonsteroidal antiestrogens, anti-hypertensives, beta blockers, beta-2-adrenergic stimulants, beta-2-receptor agonists, diuretics, vasodilators, statins, adrenaline derivatives, tocolytic drugs used to stop premature labor, selective bronchodilators and anti-asthma medications, coughing inhibitors, a wide array of amphetamines, painkillers, stimulants, appetite inhibitors, and even cocaine (Roiffé et al., 2019; Hallak, 2020).

Another relevant aspect is that AAS users often utilize other PIEDs during the AAS cycle, such as growth hormone (GH) (21% of the users), clenbuterol (19%), and thyroid hormone (15%) (Smit et al., 2020), which can be combined with other drugs to prevent side effects, including human chorionic gonadotropin (hCG) (26%), tamoxifen (23%) and anastrozole (22%) (Smit et al., 2020). More recently traces of AAS were also found in e-cigarettes, which contains nicotine and a variety of other toxic substances (Harries et al., 2024). Additionally, AAS has been also used in combination with Gamma-hydroxybutyrate (GHB) for enhancing not only athletic but also sexual performance (Giorgetti et al., 2022). Therefore, one may argue that some polypharmacy practices could partially counteract the AAS-induced male infertility and dysfunction. For example, spermatogenesis could still occur with hCG usage despite undetectable FSH and LH circulating levels (Smit et al., 2020; Bond et al., 2022). Specifically, hCG potentially preserves spermatogenesis during gonadotropin suppression (Matsumoto et al., 1986). As demonstrated here, it is highly challenging to investigate the overall effects of polypharmacy and the use of unknown or hidden substances, so its potential anecdotal benefits and risks remain largely unknown. Therefore, the real-world of AAS abusers is highly complex, making it challenging to elaborate evidence-based recommendations for the prevention and management of AAS adverse events.

## 3 Anabolic androgenic steroids and male sexual dysfunction

From a male sexual perspective, it is known that AAS abuse elicits a wide range of side effects, which may be totally or partially reversible after AAS use cessation. For instance, a recent meta-analysis, which included 24 studies and a total of 2,411 participants, on the consequences of AAS abuse on sexual parameters found that up to 44% reported reduced testicular volume, 31% had decreased libido, and 19% had erectile dysfunction (Corona et al., 2022). These results are corroborated by a recent online survey of male AAS users located in the United Kingdom, which revealed that testicular atrophy was the most reported physiological side effect noticed by the responders (Grant et al., 2023). A prospective cohort of 100 AAS users uncovered a high prevalence of sexual side effects, since there was a high prevalence of testicular atrophy (71% of participants), decreased libido (58%), erectile dysfunction (38%), priapism (24%), and subfertility (8%), whereas only 3% reported no side effects (Smit et al., 2020). Additionally, post-cycle therapy (PCT) was reported by 80% of the participants (Smit et al., 2020), which aimed to minimize the chances of AAS side effects and accelerate HPG-axis functionality recovery after the AAS cycle. Notably, the sexual side effects of AAS abuse can also be persistent or even emerge after the AAS cessation. For example, in an online survey mentioned earlier (Grant et al., 2023), it was found that 95% of the responders reported at least one symptom upon AAS cessation, with 57% reporting reduced sex drive, and 52% worrying about their recovery of testosterone and fertility. Additionally, about 56% reported using PCT to mitigate residual side effects of AAS (Grant et al., 2023). Thus, based on recent data, it could be suggested that male sexual dysfunction is widely common among AAS abusers, but little attention has been given to this issue.

## 4 Anabolic androgenic steroids and male infertility

Infertility corresponds to the failure in achieving a successful pregnancy after 12 months or more of regular unprotected intercourse (World Health Oganization, 2021). Male-factor infertility is estimated to be the cause of not less than 50% of all infertile couples (de Souza and Hallak, 2011). In fact, previous publication estimated that male infertility in Latin America reached 52% of the couples with time-to-pregnancy higher than 12 months of unprotected and frequent coitus (Agarwal et al., 2015). Traditional semen parameters are of clinically significant value in the diagnosis of male infertility provided that appropriate quality control and assurance programs are in operation, performed preferentially by andrology laboratories. Of notice, semen analysis does not provide and differentiate between a fertile and an infertile male, since fertility is a binomial condition based on two individuals, the male and the female counterpart. Nonetheless, comprehensive high quality semen analysis is a cost-effective and essential tool in the investigation of the male´s fertility potential and to stablish decision limits for further evaluation and treatment regimes. The World Health Organization (WHO) 6th edition manual for the examination and processing of human semen launched in 2021 (World Health Oganization, 2021), stablished the lower limits for semen analysis. The 5th percentile range are, sperm concentration: 16 million sperm/mL (15–18); total sperm number per ejaculate: 39 million (35–40); total motility: 42% (40–43); progressive motility: 30% (29–31); normal forms 4% (3.9–4.0). Oligozoospermia can be defined as less than 16 million spermatozoa per milliliter, moderate oligozoospermia as less than 5 million per milliliter and severe oligozoospermia refers to a meager number of sperm, typically less than 1 million per milliliter of the ejaculated volume. Azoospermia can be defined as the complete absence of spermatozoa in the ejaculate. Unfortunately, due to a lack of accurate data in the literature, it is currently not possible to estimate the prevalence of azoospermia, oligozoospermia, and severe oligospermia in male AAS users; however, studies on male contraceptive therapies may shed some light on the efficacy of testosterone intake and reduced sperm count. Briefly, the study of male infertility has been dated from the early 1990s, when Matsumoto (1990) reported that 6 months of supraphysiological doses of testosterone (i.e., ≥100 mg per week) drastically suppressed sperm counts. That study also revealed that even smaller doses of testosterone (i.e., 25 and 50 mg per week) also negatively affected sperm counts in young males (Matsumoto, 1990). More recently, studies on male contraception therapies showed a high impact of exogenous testosterone doses on reducing sperm counts (Gu et al., 2009; Gu et al., 2003), as one study found ∼95% of treatment efficacy in inducing azoospermia or severe oligozoospermia within the 6-month treatment period (Gu et al., 2009). A recent narrative review, which utilized data from five controlled randomized trials, provided an estimated time course of male infertility according to the type and dose of testosterone utilized, in which the time to reach azoospermia or oligospermia varied between 90 and 180 days, respectively. Specifically, whereas one study exhibited reduced sperm counts within 90 days following the use of AAS (i.e., 200 mg enanthate weekly doses) (World Health Organization, 1996), another study found reduced sperm counts within 180 days (i.e., 100–300 mg enanthate weekly doses) (Matsumoto, 1990). Thus, exogenous testosterone could be highly effective to induce and maintain male infertility. Although precise data are scant, azoospermia, oligozoospermia, and severe oligospermia are potentially prevalent conditions among AAS users. Recently, a retrospective cohort study aimed to investigate male fertility before and after AAS abuse (Windfeld-Mathiasen et al., 2021) in fitness centers where participants underwent an anti-doping program. The participants received a two-year ban from all fitness centers enrolled in the study in case of either a positive test for AAS usage or if the participant refused to provide a urine sample. Thus, the researchers were able to track the birth rate (i.e., from the live births documented in the Danish Medical Birth Registry) from two groups: 1) banned participants (*n* = 545) and 2) control (i.e., 10 participants, matched by years of age, for every banned participant) for 10 years before and after the suspension period. The main findings were: 1) when analyzing the 10 years before the sanction period, the banned participants had 26% lower fertility rate compared to the control, and 2) after completing the follow-up analysis, the banned participants had only 6% lower fertility rate compared to controls. The authors found a 123% increase in fertility rate 3 years after the suspension period among the banned participants compared to a 14% increase in the control group. These data reinforce the negative (although partially transitory) effects of AAS abuse on male fertility.

In the context of male infertility and AAS abuse, data from the HAARLEM study are highly informative as AAS abusers underwent a health screening before, at the end of AAS cycle (i.e., within the last week of AAS cycle), 3 and 12 months after the end of a AAS cycle (Smit, Buijs, de Hon, et al., 2021). The study involved 100 participants, in which 37 participants presented gonadal dysfunction at baseline (i.e., based on low testosterone level or low total sperm count [TSC]), probably due to a previous history of AAS abuse. At the end of the AAS cycle, all participants decreased their testicular volume (average of −4.3 mL) and TSC (average loss of 120 million), with 66% being diagnosed either with oligo- or azoospermia and 77% having TSC below 40 million. Additionally, all participants exhibited undetectable LH and FSH and increased testosterone levels, whose changes were directly correlated to the total cycle duration and total AAS used (in mg). During the recovery period, whereas testosterone and LH recovered 3 months after the AAS cycle, the testicular volume, TSC, and semen concentration remained below baseline values. Importantly, semen volume and TSC were still lower than baseline values even after 12 months following the AAS cycle cessation. Another fascinating insight was that among participants with gonadal dysfunction at baseline, TSC recovery took a longer time compared to first-time AAS users with normal gonadal function (69 weeks vs. 56 weeks, respectively).

To date, the decrease in male fertility is thought to be due to the negative feedback of androgens on the HPG axis, thus diminishing spermatogenesis and, in the long run, decreasing testicular volume. Of note, even though the circulating testosterone levels are at supraphysiological concentrations during the AAS cycle, the intratesticular testosterone concentration lowers due to the hypogonadotropic state induced by AAS usage (Sidhom et al., 2022), which diminishes the testosterone production via the Leydig cells (Sidhom et al., 2022). Specifically, the Leydig cells under the AAS abuse effect might decrease their population because of the AAS-induced hypogonadotropic state (Feinberg et al., 1997). Additionally, the decrease in endogenous FSH production could reduce testicular cells’ testosterone uptake since FSH induces androgen-binding protein production, which is responsible for sequestering testosterone in the testis (Hall et al., 1990). In parallel, testosterone levels are closely related to erectile function (Isidori et al., 2014), which can be defined as the ability to achieve or maintain an erection sufficient for satisfactory sexual performance (NIH Consensus, 1993). Erectile dysfunction can be caused by psychogenic and/or organic factors (Shamloul and Ghanem, 2013). Specifically, erectile dysfunction in AAS users is mostly evidenced post-cycle since the testosterone level considerably diminishes, and the user could feel some adverse psychological effects, such as symptoms of depression and decreased libido (Bond et al., 2022). Noteworthy, the erectile dysfunction post-AAS cycle could be boosted by the sharp increase in libido during the cycle. In rare occasions, erectile dysfunction can be diagnosed during the AAS cycle, in this case, driven mainly by organic factors since oestradiol also plays a role in erectile function (Finkelstein et al., 2013), and it could involve an imbalance between androgenic and estrogenic action. Erectile dysfunction during the AAS cycle could also be a consequence of the sharp increase in libido, thus hindering a healthy and mutual sexual relationship with the sexual partner. To date, whereas there is no evidence of histological or molecular changes associated with erection, studies on penile strips have suggested an overall, possible positive effect of testosterone administration since it facilitates acetylcholine-induced relaxation and decreased contractile response to adrenergic stimulation (Corona et al., 2022). However, the expected benefit of testosterone on penile erection was not evidenced in animal studies since there was a decrease in contractile response to phenylephrine exposure and no increase in erectile function (as assessed by intracavernous pressure divided by the mean arterial pressure when the cavernous nerve was stimulated at different frequencies). Additionally, supraphysiological doses of testosterone were able to decrease sperm number and deteriorate sperm morphology without affecting its motility (Corona et al., 2022).

## 5 Potential treatments

Despite the growing evidence of the adverse effects of AAS abuse on male fertility and sexuality, less attention has been given to potential treatment strategies (Anawalt, 2019). Only recently, protocols have been developed to guide health screening exams (van de Ven et al., 2020), harm reduction strategies (Eu et al., 2023), and pharmacological interventions to restore fertility (Rizzuti et al., 2023). A recent task force from various Australian institutions has released a summary of potential exams that should be considered according to the pattern and timing of the AAS cycle. For example, neuroendocrine blood analysis (i.e., testosterone, LH, FSH, SHBG, IGF-1, TSH, and PSA) is more helpful around 3 months after AAS use cessation, when hormone levels could be considerably altered (van de Ven et al., 2020). Additionally, semen analysis should preferably be done after 6 months of AAS cessation since spermatogenesis takes longer to show signs of recovery (van de Ven et al., 2020), as previously discussed herein. The reader is referred to for further guidance on clinical and biochemical exams (van de Ven et al., 2020).

Notably, there is a lack of data on harm reduction (Anawalt, 2019; Bonnecaze et al., 2021), which are strategies that aim to reduce and cease the use of AAS in the long term. However, it is worth noting that stopping the use of AAS is complex (Pope, Wood, et al., 2014b), as most users are affected by addiction and post-use withdrawal syndrome, which is characterized by symptoms of depression, a marked drop in libido and anhedonia (Pope, Wood, et al., 2014b; Anawalt, 2019). All of these side effects stimulate the continuous and recurrent use of AAS. Therefore, instead of ceasing the use of AAS, harm reduction plays the role of guiding the user to 1) reduce the frequency and dose of AAS, 2) minimize health complications due to the misuse of additional substances and contaminated material, and 3) provide specialized multidisciplinary support for long-term monitoring of health parameters. To date, recent evidence demonstrates the beneficial effects of harm reduction through educational approaches and assistance to both users and medical doctors (Eu et al., 2023). Researchers in Australia have trained doctors from several clinics and hospitals serving AAS users in the country on harm reduction strategies that can be implemented during a medical consultation (Eu et al., 2023). The training consisted of providing information to physicians about possible adverse events, which tests and procedures should be performed, and the relevance of welcoming the patient and not judging the reasons for using AAS. After the training protocol, physicians reported that 44% of the AAS users declared their intention to reduce their use, and 12% declared their intention to cease AAS use. One of the most significant barriers in promoting harm reduction, and even cessation of AAS use, is that AAS users do not trust their physicians and, thus, very often omit their current and past AAS use (van de Ven et al., 2020; Eu et al., 2023). For this reason, it is advised to clinicians that the first step towards AAS cessation is a behavioral approach, which may comprise motivational interviewing (Bischof et al., 2021) and other validated techniques, which may be particularly relevant for those patients that show signs of infertility awareness (Rizzuti et al., 2023).

In combination with harm reduction strategies, some pharmacological approaches could potentially restore male fertility and sexual function in AAS users. There are some tested drugs with positive results (Rizzuti et al., 2023), such as selective estrogen receptor modulators (SERMs; e.g., clomiphene citrate and tamoxifen), LH receptor agonists (e.g., hCG and recombinant LH), human menopausal gonadotropin (hMG), recombinant FSH, and aromatase inhibitors (e.g., anastrozole and letrozole). These abovementioned drugs are considered adequate; however, the decision of using one or a combination of them to restore fertility should be taken according to the patient’s present or future desire to conceive or not. Moreover, one of the goals of medical treatment, should aim to restore testicular function characterized by an improvement in sperm quality and intrinsic hormonal production (Pagani et al., 2018). It must be highlighted that randomized controlled trials of pharmacological treatments for AAS-induced infertility have found mixed results. For example, Al Hashimi (2022) treated post-AAS users (i.e., at least 3 months without AAS use; *n* = 463) who reported infertility (i.e., trying to conceive for >1 year) and sexual dysfunction (i.e., erectile dysfunction, low sexual desire, defective orgasm, or ejaculation) with hCG injections and clomiphene citrate tablets intake [i.e., see Al Hashimi (2022)] for 12 months. Overall, there was a faster improvement regarding the international index of erectile function, hormone levels, testicular size, and semen parameters in treated patients compared to the control group (i.e., no treatment; *n* = 57). Conversely, other trials failed to find improvements in fertility among post-AAS users during 6 months of treatment comprising clomiphene citrate (50 mg oral intake every other day) and hCG (2,000 IU subcutaneous injections three times weekly) (Ledesma et al., 2023). Amongst the 24 participants enrolled in this study, only nine achieved successful pregnancy, three of them utilizing assisted reproductive technology. These two recent studies highlight the complexity of restoring male fertility and sexual function in AAS uses. Therefore, one reasonable option to secure future fertility potential in individuals who are willing to start the use of AAS might be to discuss sperm cryopreservation of several semen samples in case of male infertility occurs (Ranganathan et al., 2002). Sperm cryopreservation is a standardized and well-stablished laboratorial procedure with several protocols aimed at obtaining the best pos-thaw results according to the characteristic of individual characteristics of the semen sample, the patient and his underlying medical conditions (Hallak et al., 2000; Ranéa et al., 2022). A critical moment for fertility preservation is during and after andrological treatment for subfertile or infertile male, as they seek medical aid because of inability to achieve natural pregnancy or through assisted reproductive techniques, and they have restored their testicular function and improved sperm quality *in vivo* or *in vitro* (Pasqualotto et al., 2006; Pariz et al., 2019) or stored their gametes for decades, guaranteeing to father their own biological offspring (Pariz et al., 2020). Rizzuti et al. (2023) developed a decision tree based on the responses and context of the AAS user, ultimately leading to the best approach to reestablishing fertility and reducing harm. In case the patient reports the desire to have children but is not willing to stop AAS use, sperm cryopreservation is recommended since the duration and total amount of AAS use are directly correlated to infertility. Depending on the goal of the patient and his willingness to stop using AAS, other treatments are recommended, such as hGC intake of 125–250 international units (IU) every other day and follow-up with periodic visits and exams.

## 6 Limitations

The current review has some inherent limitations. First, the prevalence of adverse events is commonly estimated by using remote or in-person surveys, which might introduce bias because participants may overestimate their sexual performance or not report specific sexual and fertility issues. Additionally, sexual dysfunction and infertility prevalence estimates could be inaccurate when based solely in questionnaires, that is, without clinical confirmation (Fenton, 2001). Second, and more importantly, in contrast to systematic reviews, a narrative review is subjected to limitations related to the authors’ subjectivity in the choice of literature, completeness of literature search, and interpretation of the main findings (Green et al., 2006; Sukhera, 2022).

## 7 Gaps in literature, concluding remarks and future directions

The investigation of the abusive use of AAS is not straightforward since studies with randomized controlled designs are impeded by the obvious ethical implications of prescribing such substances at supraphysiological doses to healthy individuals. Replicating within a controlled trial the variety of cycles and combinations of drugs seen in the real world is neither feasible from a methodological perspective nor ethically acceptable. Thus, most findings on the effects of abusive use of AAS have been deemed as of low scientific quality since they mainly come from cross-sectional studies, retrospective studies, case studies, and opinion articles.

To partially overcome the limitations in the literature, further studies like the HAARLEM’s, adopting prospective cohort designs and minimally interfering with the customary practices of AAS users might be a wise alternative. It is also crucial that novel studies comprise many individuals beyond young male adults. Data suggest that AAS use typically begins between 15 and 30 years of age (Pope, Kanayama, et al., 2014). Some studies suggest that adolescents should be the focus of educational policies and harm reduction interventions, given that there is a high prevalence of addiction in this age group, and the use of AAS tends to increase throughout life (Sagoe et al., 2014; Kanayama and Pope, 2018). Nonetheless, it is currently unknown what the effects of AAS abuse on fertility and sexual function would be throughout puberty since the hormonal changes are at the most relevant phase for male sex maturation (de Souza and Hallak, 2011). Likewise, little is known about the effects of AAS in older adults. Efforts should also be made to test harm reduction strategies to prevent AAS-induced damage, as well as new pharmacological treatments for the recovery of fertility and sexual dysfunction. Identifying the most vulnerable patients and the most harmful drugs (and their combination) able to induce these adverse events is also desirable. Moreover, viability and adequacy studies on public health policies and educational programs are warranted to reduce AAS’s mis-prescription and abusive use. Finally, to increase the knowledge regarding the physiological mechanistic basis of male infertility under AAS abuse, future investigations should include more sophisticated techniques such as semen oxidation-reduction tests like oxidative stress (Cocuzza et al., 2008), reactive oxygen species (Athayde et al., 2007), reactive nitrogen species, lipid peroxidation (Drevet et al., 2022), DNA damage (Hallak, 2017), mitochondrial activity (Pariz and Hallak, 2016), and determination of extracellular neutrophil traps.

Although ethical issues indeed hamper more controlled studies on this topic, one should note that, owing to similar reasons, there is a lack of controlled trials for other well-known risk factors, such as smoking and alcohol intake, which did not preclude the science from accumulating several levels of evidence that are now used to tailor prevention policies and programs. Therefore, despite the gaps in the literature, existing data allows the conclusion that AAS abusers are more susceptible to infertility and sexual dysfunction. Although these adverse effects may be transitory in some individuals, it is unclear who is more vulnerable to permanent harmful effects, nor is it known if the existing therapy can fully resolve these conditions in the long run. In this context, efforts to prevent the abusive use of these substances should be implemented from clinical to public health levels.
